# Preventing Alveolar Osteitis After Molar Extraction Using Chlorhexidine Rinse and Gel: A Meta-Analysis of Randomized Controlled Trials

**DOI:** 10.1097/jnr.0000000000000401

**Published:** 2020-09-21

**Authors:** Chia-Hui WANG, Shu-Hui YANG, Hsiu-Ju JEN, Jui-Chen TSAI, Hsi-Kuei LIN, El-Wui LOH

**Affiliations:** 1 PhD, RN, Supervisor, Department of Nursing, Taipei Medical University Shuang Ho Hospital, and Assistant Professor, School of Nursing, College of Nursing, Taipei Medical University, Taiwan, ROC; 2 BSN, RN, Department of Nursing, Taipei Medical University Shuang Ho Hospital, Taiwan, ROC; 3 BSN, RN, Head Nurse, Department of Nursing, Taipei Medical University Shuang Ho Hospital, and Adjunct Assistant Professor, School of Nursing, College of Nursing, Taipei Medical University, Taiwan, ROC; 4 MSN, RN, Consultant, Department of Nursing, Taipei Medical University Shuang Ho Hospital, and Adjunct Assistant Professor, School of Nursing, College of Nursing, Taipei Medical University, Taiwan, ROC; 5 DDS, Department of Dentistry, Taipei Medical University Shuang Ho Hospital, and Lecturer, School of Dentistry, College of Oral Medicine, Taipei Medical University, Taiwan, ROC; 6 PhD, Joint Appointment Medical Researcher, Center for Evidence-Based Health Care and Shared Decision Making Resource Center, Department of Medical Research, and Department of Dentistry, Taipei Medical University Shuang Ho Hospital; Researcher, Cochrane Taiwan, Taipei Medical University; and Assistant Professor, Graduate Institute of Clinical Medicine, College of Medicine, Taipei Medical University, Taiwan, ROC; 7 contributed equally.

**Keywords:** chlorhexidine, rinse, gel, alveolar osteitis

## Abstract

**Background:**

Alveolar osteitis (AO) may occur after molar extraction. Chlorhexidine (CHX) rinse and CHX gel are widely used to prevent AO. Although previous meta-analyses support the effectiveness of both CHX rinse and CHX gel in preventing AO, important issues regarding these two formulations have not been addressed adequately in the literature.

**Purpose:**

A systematic review and meta-analysis of randomized controlled trials was conducted to determine the effectiveness of CHX rinse and CHX gel in preventing AO.

**Methods:**

PubMed, EMBASE, SCOPUS, and Cochrane databases were searched for randomized controlled trials published before June 2018. The risk ratio (RR) was used to estimate the pooled effect of AO incidence using a random-effect model.

**Results:**

The RRs of AO in patients treated with 0.12% CHX rinse (RR = 0.54, 95% CI [0.41, 0.72]) and 0.2% CHX rinse (RR = 0.84, 95% CI [0.52, 1.35]) were significantly lower than in those treated with the control. Moreover, a significantly lower RR was identified in patients treated with 0.2% CHX gel (RR = 0.47, 95% CI [0.34, 0.64]) than in those treated with the control. When CHX products of different concentrations were grouped together, patients treated with CHX rinse showed an RR of AO of 0.61 (95% CI [0.48, 0.78]) and those treated with CHX gel showed an RR of AO of 0.44 (95% CI [0.43, 0.65]). On the other hand, a meta-analysis of three trials that compared CHX rinse and CHX gel directly showed a significantly lower RR of AO in patients treated with CHX rinse than in those treated with CHX gel (RR = 0.56, 95% CI [0.34, 0.96]).

**Conclusions/Implications for Practice:**

The results support the effectiveness of both CHX rinse and gel in reducing the risk of AO after molar extraction. Each formulation provides unique benefits in terms of ease of application and cost. On the basis of the results of this study, the authors recommend that CHX gel be used immediately after molar extraction because of the convenience and cost-effectiveness of this treatment and that CHX rinse be used by the patient after discharge at home in combination with appropriate health education and case management.

## Introduction

Alveolar osteitis (AO), the inflammation of the alveolar bone when an intra-alveolar blood clot disintegrates or fails to form, is one of the most common complications occurring after third molar (wisdom-tooth) extraction (Cardoso et al., 2010). AO usually manifests 2–5 days after surgery and is one of the main reasons for seeking postsurgical emergency appointments (Lee et al., 2015). Patients may experience fetid breath and persistent and radiating pain, which is not easily relieved by analgesics. The AO incidence after tooth extraction ranged from 3.2% to 6.14% in studies with large sample sizes (Abu Younis & Abu Hantash, 2011; Congiusta &Veitz-Keenan, 2013; Sigron et al., 2014) and even up to 35% in early studies (Erickson et al., 1960). Moreover, the risk of AO has been reported to be associated with the degree of difficulty involved in molar tooth extraction (Ogunlewe et al., 2007) and is often higher after surgical extractions than after nonsurgical extractions (Nusair & Younis, 2007).

A series of pharmacological agents, including antibacterial agents, antifibrinolytic agents, antiseptic agents, obtundent dressings, steroidal anti-inflammatory agents, clot-support agents, and growth-factor-rich plasma (Blum, 2002; Haraji et al., 2012), have each been examined for their potential in preventing AO. Chlorhexidine (CHX), an antiseptic agent developed in the 1940s, inhibits the growth of bacteria by increasing their cytoplasmic permeability and causing cell lysis. This agent is widely used for antibacterial purposes in hygiene control and surgery (Balagopal & Arjunkumar, 2013). CHX is available in the market in the form of different hygiene and treatment products such as chewing gum, toothpaste, spray, rinse, gel, and varnishes. Previous meta-analyses on both the gel and rinse formulations (Caso et al., 2005; Yengopal & Mickenautsch, 2012; Zhou et al., 2017) have suggested a prophylactic effect of CHX in terms of reducing the risk of AO after molar extraction. Although a recent systematic review and meta-analysis provided additional information on this issue, the focus of the review was on specific types of formulation (Dobson et al., 2018; Teshome, 2017) and more-recent publications were not covered (Rodriguez Sanchez et al., 2017).

A new meta-analysis of randomized controlled trials (RCTs) was performed in this study to evaluate the effectiveness of CHX rinse and CHX gel, the two most common types of CHX prophylactics, in preventing AO after molar extraction.

## Methods

### Literature Search

A search of the PubMed, EMBASE, SCOPUS, and Cochrane databases up to June 2018 was conducted to identify relevant trials. The following MeSH search headings were used: “alveolar osteitis,” “dry socket,” “alveolitis sicca dolorosa,” “fibrinolytic alveolitis,” “chlorhexidine,” “CHX,” “molar extraction,” “extraction,” “molar removal,” “molar surgery,” and “surgery.” These terms and their combinations were searched as text words. The “related articles” function in PubMed was used to broaden the scope of search. All of the abstracts, studies, and citations retrieved in this search were reviewed. In addition, other trials identified by manually searching the reference sections of the accessed articles and by contacting known experts in the field were reviewed. Furthermore, relevant unpublished trials registered on the ClinicalTrials.gov registry (http://clinicaltrials.gov/) and otherwise-unlisted articles searchable in Google Scholar were searched and reviewed as well. No language restrictions were applied.

### Trial Selection

Trials that met the following criteria were included in the analysis: evaluating the efficacy of CHX rinse or gel in preventing AO in dental patients undergoing molar extraction or surgery, clearly stating the inclusion and exclusion criteria used to select patients for participation, and adequately describing the molar extraction or surgical procedures. Trials or data were excluded from analysis that examined additional components such as active gel-containing CHX and metronidazole versus placebo gel that would confound the contribution of CHX or that compared the efficacy of different CHX implication protocols of dosage, administration time, and treatment period. When duplicated articles with overlapping data sets were identified, the trial with the larger population was included.

### Data Extraction and Quality Assessment

Two reviewers independently extracted the following information from each trial: first author, year of publication, trial population characteristics, trial design, inclusion and exclusion criteria, matching criteria, definition of molar tooth extraction, and incidence of AO. The retrieved studies were assessed for eligibility by the two reviewers according to the specified inclusion criteria. The individually recorded decisions of the two reviewers were compared, and any disagreements were resolved by a third reviewer.

The quality of the retrieved trials was assessed using the revised Cochrane risk-of-bias tool for randomized trials Version 2 recommended by the Cochrane Collaboration (Higgins et al., 2019). Two reviewers conducted the assessment, and any disagreement was resolved by a third reviewer. Five domains were assessed, including bias arising from the randomization process, bias due to deviations from intended interventions (effect adhering to intervention), bias due to missing outcome data, bias in measuring outcomes, and bias in selecting the reported results.

### Data Synthesis and Analysis

The statistical package Review Manager, Version 5.3 (Cochrane Collaboration, Oxford, England) was used to analyze the data. The meta-analysis was performed according to the recommendations in the Preferred Reporting Items for Systematic Reviews and Meta-Analyses guidelines (Moher et al., 2009). When necessary, standard deviations were estimated using the provided confidence interval (CI) limits, standard error, or range values.

Data were pooled only for trials that reported sufficiently similar clinical and methodological variables. A pooled estimate of risk ratio (RR) was computed using the DerSimonian and Laird random-effect model (DerSimonian & Laird, 1986). Heterogeneity among the trials was assessed using the *I*^2^ test and a null hypothesis test, in which *p* < .1 was considered to represent significant outcome heterogeneity. Because the included trials used CHX in rinse or gel formulations at different CHX concentrations, subgroup analyses were conducted according to their packing materials (rinse or gel) and CHX concentrations to examine differences attributable to differences in composition.

## Results

### Trial Characteristics

**Figure 1.**
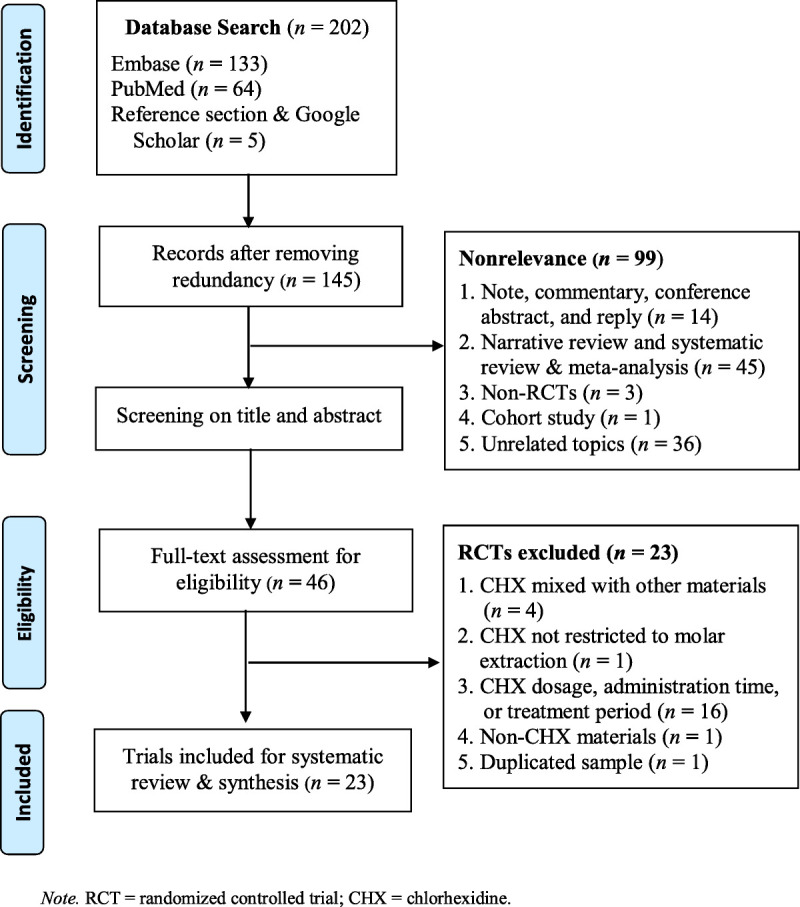
Sampling Procedures

The procedures used in sampling are summarized in Figure 1. The initial search yielded 202 records, of which 145 records were excluded because of duplication. After a brief reading of the title and abstract, a further 99 records, including 14 short publications, 45 reviews (narrative review and systematic review and meta-analysis), three non-RCTs, one cohort study, and 36 dentistry articles, were excluded because of lack of relevance to the scope of this review. The full contents of the remaining 46 records were retrieved for evaluation. Subsequently, 23 records were excluded, including four RCTs that tested CHX mixed with other materials; one RCT that was not restricted to molar extraction; 16 RCTs that tested CHX dosage, administration time, or treatment period; and one RCT that used a duplicate sample. Of the remaining 23 trials, 20 were identified from the academic databases and three (Ahmedi et al., 2014; Shaban et al., 2014; Younus et al., 2014) were identified through Google Scholar. The data from these 23 trials were used in analysis, and their characteristics are presented in Table 1. The included studies were published between 1991 and 2017 and had sample sizes of 30–271 patients. Seven of the trials examined the effectiveness of CHX rinse, 13 examined the effectiveness of CHX gel, and three examined the comparative effectiveness of CHX rinse and CHX gel. In terms of controls, a subject–subject case–control design was used in 18 of the trials, and a split-mouth case–control design was used in five of the trials. All of the included trials restricted their subjects to single third molar or bilateral third molar extractions with the exceptions of Khan et al. (2015) and Abu-Mostafa et al. (2015), which allowed the extraction of any molar tooth.

**Table 1.** Characteristics of the Selected Randomized Controlled Trials

Trial/Author (Year)	Inclusion Criteria	No. of Patients (% Male)	Mean Age or Mean Age ± *SD*	Treatment
Rinse vs. control					
1. Berwick & Lessin (1990)	Bilateral max. third molars	I: 20 (NA) C: 20 (NA)	21.4	I: 0.12% CHX rinse for 1 min before surgery C: NSR after surgery	
2. Channar et al. (2013)	Mand. third molar	I: 73 (NA) C: 72 (NA)	30.4 ± 5.2	I: 0.2% CHX rinse (15 ml for 30 s), twice daily for 7 days after surgery C: NSR	
3. Delilbasi et al. (2002)	Mand. third molar	I: 62 (48.0) C: 59 (42.4)	I: 24.1 C: 24.2	I: 0.2% CHX rinse (15 ml for 30 s) before surgery and twice daily for 7 days after surgery C: NSR before surgery and twice daily for 7 days after surgery	
4. Hermesch et al. (1998)	≥ 1 Mand. third molar	I: 136 (37.5) C: 135 (37.0)	I: 22.2 C: 22.4	I: 0.12% CHX rinse (15 ml for 30 s), twice daily for 7 days before and 7 days after surgery C: 11.6% alcohol (15 ml for 30 s), twice daily for 7 days before and 7 days after surgery	
5. Larsen (1991)	Bilateral mand. third molars	I: 73 (43.8) C: 67 (44.8)	NA	I: 0.12% CHX rinse (15 ml for 30 s), twice daily for 7 days before and 7 days after surgery C: Identical solution without CHX (15 m; for 30 s), twice daily for 7 days before and 7 days after surgery	
6. Osunde et al. (2017)	Mand. third molars	I: 50 (48) C: 50 (44)	I: 26.4 ± 5.1 C: 27.1 ± 5.9	I: Gargle with 0.12% CHX gluconate rinse twice daily C: Gargle with warm NSR twice daily	
7. Ragno & Szkutnik (1991)	Mand. third molars	I: 80 C: 80	NA	I: 0.12% CHX rinse. Rinse with 15 ml before suture and then the day after surgery (15 ml for 30 s), twice daily for 7 days C: Placebo. Rinse with 15-ml placebo solution before suture and then the day after surgery (15 ml for 30 s), twice daily for 7 days	
Gel vs. control					
8. Ahmedi et al. (2014)	Bilateral mand. third molars	I: 25 C: 25	NA	I: 2-ml 1% CHX digluconate gel into alveolus before suture C: NSR of alveolus before suture	
9. Babar et al. (2012)	Mand. third molar	I: 50 (78) C: 50 (54)	29 ± 6	I: 0.2% CHX gel into alveolus C: No treatment	
10. Freudenthal et al. (2015)	Mand. third molars	I: 48 (51) C: 47 (44)	I: 33 C: 34	I: 10-ml Cervitec gel (0.2% CHX and 0.2 sodium fluoride) into alveolus before suture C: 10-ml placebo gel (0.2% sodium fluoride) into alveolus before suture	
11. Haraji et al. (2013)	Bilateral mand. third molars	I: 80 (48.8) C: 80 (48.8)	21.6 ± 2.5	I: 0.2% CHX gel into alveolus before suture C: Dry dressing into alveolus before suture	
12. Haraji & Rakhshan (2014)	Bilateral mand. third molars	I: 45 (53.3) C: 45 (53.3)	22.1 ± 2.7	I: 0.2% CHX gel into alveolus before suture C: Dry dressing into alveolus before suture	
13. Jesudasan et al. (2015)	Mand. third molars	I: 90 C: 90	I: 28 ± 6 C: 28 ± 7	I: 0.2% CHX gel into alveolus before suture C: No treatment before suture	
14. Khan et al. (2015)	Max. or mand. first, second, or third molar	I: 128 C: 125	36.7 ± 11.0	I: Bite on gauze with 10-ml 0.2% CHX gluconate gel for 15 min C: Bite on gauze with placebo gel for 15 min	
15. Requena-Calla & Funes-Rumiche (2016)	Mand. third molar	I: 20 (50) C: 20 (65)	I: 23.1 C: 22.9	I: 1-ml 0.12% CHX gel into alveolus before suture C: 1-ml placebo gel into alveolus before suture	
16. Rubio-Palau et al. (2015)	Mand. third molars	I: 80 (48.7) C: 80 (51.7)	25.04	I: 10-ml 0.2% CHX bioadhesive gel in alveolus C: 10-ml placebo gel in alveolus	
17. Shaban et al. (2014)	Bilateral mand. third molars	I: 41 (34.1) C: 42 (31.1)	24.2 ± 5.0	I: 0.2% CHX gel into alveolus C: No treatment
18. Torres-Lagares, Infante-Cossio, et al. (2006)	Mand. third molar	I: 17 (29.4) C: 13 (30.8)	I: 29 ± 10.2 C: 26.3 ± 6.0	I: 0.2% CHX adhesive gel into alveolus C: No treatment
19. Torres-Lagares, Gutierrez-Perez, et al. (2006)	Mand. third molar	I: 53 (37.7) C: 50 (28.0)	I: 27.8 ± 8.4 C: 25.7 ± 8.6	I: 0.2% CHX digluconate bioadhesive gel into alveolus C: Placebo gel into alveolus
20. Torres-Lagares et al. (2010)	Mand. third molar	I: 24 (78.6) C: 14 (91.7)	I: 32.5 ± 16.7 C: 32.0 ± 11.9	I: 10-ml 0.2% CHX bioadhesive gel in alveolus C: 10-ml placebo gel into alveolus
Rinse vs. gel				
21. Abu-Mostafa et al. (2015)	Max. or mand. molar	G: 160 R: 141	G: NA R: NA	G: 0.2% CHX bioadhesive gel into alveolus on the first and third day after surgery R: 0.12% CHX rinse from the second day, twice daily for 7 days
22. Hita-Iglesias et al. (2008)	Mand. third molar	G: 41 (34.2) R: 32 (15.6)	G: 28 R: 26	I: 0.2% CHX bioadhesive gel on wound, twice daily for 7 days C: 0.12% CHX rinse, twice daily for 7 days
23. Younus et al. (2014)	Mand. third molar	G: 50 (58) R: 50 (62)	G: 23.5 ± 5.1 R: 22.9 ± 5.2	G: 0.2% CHX gel in alveolus, 4 times daily for 7 days R: 10-ml 0.2% CHX rinse, 4 times daily for 7 days

***Note.*** I = intervention group; C = control group; NA = not available; CHX = chlorhexidine; NSR = normal saline rinse; Mand. = mandibular; Max. = maxillary; min = minute; NaF = sodium fluoride; G = gel group; R = rinse group; s = seconds.

The methodological quality of the eligible trials is presented in Table 2. All of the included trials reflected low risk of bias because of either deviations from intended interventions or missing outcome data. The low risk is likely attributable to the high level of motivation that the participants had in reducing postoperative pain and in returning for follow-up examinations. However, all of the included trials reflected some level of concern with regard to bias in reporting results, as none mentioned the registration of their protocols. Most of the included trials had either a low risk or some concerns for bias related to the randomization process, although one trial that compared CHX gel and the control and three trials that compared CHX rinse and CHX gel were found to reflect a high risk of bias in the domain. The participants in those trials that compared CHX rinse and CHX gel would know their allocation sequences even if the allocation was random. Furthermore, the one trial that compared CHX gel and the control had a high risk of bias in randomization process because no treatment was provided to the controlled subjects. Finally, there was a mixture of classes of bias in terms of outcome measurement, with 13 deemed at a low risk, six deemed as having some concerns, and four deemed at a high risk.

**Table 2.** Assessment of the Methodological Quality of Selected Trials Using the Revised Cochrane Risk-of-Bias Tool for Randomized Trials (Version 2)

Trial	Randomisation	Deviation From Intended Interventions	Missing Outcome	Measurement of Outcome	Reporting	Overall
CHX Rinse vs. control						
1. Berwick & Lessin (1990)	LR	LR	LR	LR	SC	SC
2. Channar et al. (2013)	SC	LR	LR	LR	SC	SC
3. Delilbasi et al. (2002)	LR	LR	LR	LR	SC	SC
4. Hermesch et al. (1998)	LR	LR	LR	SC	SC	SC
5. Larsen (1991)	LR	LR	LR	SC	SC	SC
6. Osunde et al. (2017)	LR	LR	LR	LR	SC	SC
7. Ragno & Szkutnik (1991)	LR	LR	LR	LR	SC	SC
CHX Gel vs. control						
8. Ahmedi et al. (2014)	LR	LR	LR	HR	SC	HR
9. Babar et al. (2012)	SC	LR	LR	HR	SC	HR
10. Freudenthal et al. (2015)	LR	LR	LR	LR	SC	SC
11. Haraji et al. (2013)	SC	LR	LR	LR	SC	SC
12. Haraji & Rakhshan (2014)	SC	LR	LR	LR	SC	SC
13. Jesudasan et al. (2015)	SC	LR	LR	LR	SC	SC
14. Khan et al. (2015)	SC	LR	LR	SC	SC	SC
15. Requena-Calla & Funes-Rumiche (2016)	SC	LR	LR	HR	SC	HR
16. Rubio-Palau et al. (2015)	SC	LR	LR	SC	SC	SC
17. Shaban et al. (2014)	HR	LR	LR	LR	SC	HR
18. Torres-Lagares, Infante-Cossio, et al. (2006)	SC	LR	LR	SC	SC	SC
19. Torres-Lagares, Gutierrez-Perez, et al. (2006)	LR	LR	LR	LR	SC	SC
20. Torres-Lagares et al. (2010)	SC	LR	LR	LR	SC	SC
CHX Rinse vs. gel						
21. Abu-Mostafa et al. (2015)	HR	LR	LR	SC	SC	HR
22. Hita-Iglesias et al. (2008)	HR	LR	LR	HR	SC	HR
23. Younus et al. (2014)	HR	LR	LR	LR	SC	HR

***Note.*** LR = low risk; SC = some concerns; HR = high risk.

### Incidence of Alveolar Osteitis

#### Chlorhexidine rinse versus control

**Figure 2.**
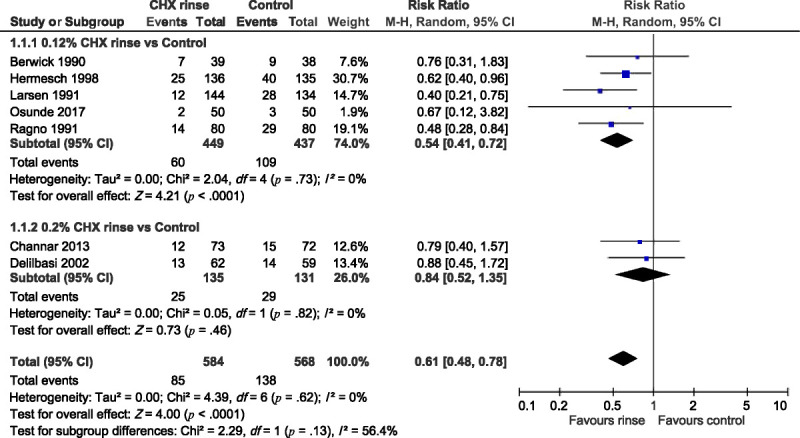
Forest Plot of Chlorhexidine Rinse (0.12% and 0.2%, Separately) Versus Control for Risk Ratio of Alveolar Osteitis

Five of the included trials compared the incidence of AO between a 0.12% CHX rinse group and a control group (Berwick & Lessin, 1990; Hermesch et al., 1998; Larsen, 1991; Osunde et al., 2017; Ragno & Szkutnik, 1991), and two trials compared the incidence of AO between a 0.2% CHX rinse group and a control group (Channar et al., 2013, Delilbasi et al., 2002). These trials were analyzed in two subgroups, with the results presented in Figure 2. The RR of AO in patients treated with 0.12% CHX rinse was significantly lower than that in patients treated with the control (RR = 0.54, 95% CI [0.41, 0.72]), with no heterogeneity across trials (*I*^2^ = 0%, *p* = .73). Furthermore, the RR of AO in patients treated with 0.2% CHX rinse was significantly lower than that in patients treated with the control (RR = 0.84, 95% CI [0.52, 1.35]), with no significant heterogeneity across trials (*I*^2^ = 0%, *p* = .82). The overall RR of both CHX types was 0.61 (95% CI [0.48, 0.78]), with no subgroup difference (*I*^2^ = 56.4%, *p* = .13) and significant heterogeneity across trials (*I*^2^ = 0%, *p* = .62).

#### Chlorhexidine gel versus control

**Figure 3.**
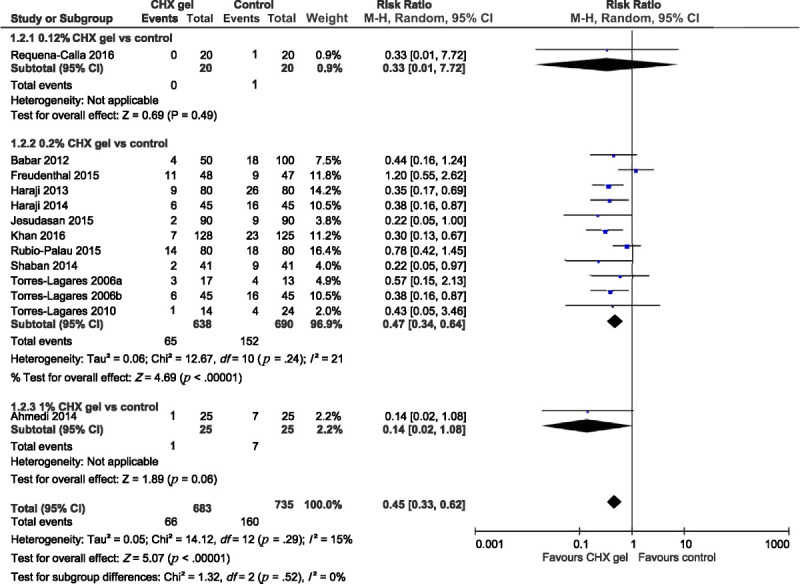
Forest Plot of Chlorhexidine Gel (0.12%, 0.2%, and 1%, Separately) Versus Control for Risk Ratio of Alveolar Osteitis

Thirteen of the included trials compared the incidence of AO between CHX gel and a control. Of these, 11 used 0.2% CHX gel, one used 0.12% CHX gel (Requena-Calla & Funes-Rumiche, 2016), and one used 1% CHX gel (Ahmedi et al., 2014). These trials were analyzed in three subgroups, with the results summarized in Figure 3. Patients treated with 0.12% CHX gel in the single trial did not show a difference compared with the control (RR = 0.33, 95% CI [0.01, 7.72]). However, patients treated with 0.2% CHX gel exhibited a significantly lower RR of AO compared with those treated with the control (RR = 0.47, 95% CI [0.34, 0.64]), with insignificant heterogeneity across trials (*I*^2^ = 21%, *p* = .24). Furthermore, patients treated with 1% CHX gel in the other single trial exhibited a lower risk of AO than those treated with the control, with borderline significance (RR = 0.14, 95% CI [0.02, 1.08]). The total effect for all of the trials included in the analysis was significant (RR = 0.45, 95% CI [0.33, 0.62]), with insignificant heterogeneity (*I*^2^ = 15%, *p* = .29) and differences between subgroups (*I*^2^ = 0%, *p* = .52) across all trials.

#### Subgroup difference

**Figure 4.**
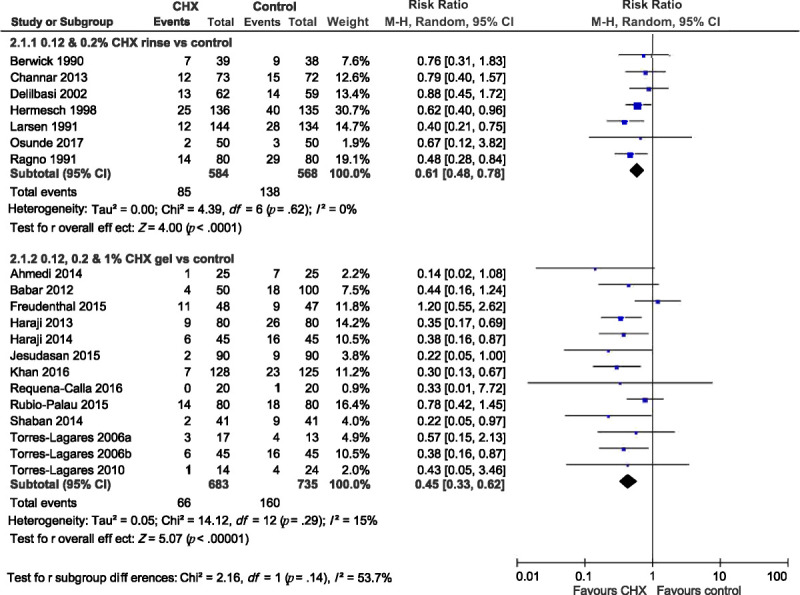
Subgroup Difference Between All Types of Chlorhexidine Rinse (0.12% and 0.2%) and All Types of Chlorhexidine Gel (0.12%, 0.2%, and 1%) for Risk Ratio of Alveolar Osteitis

To examine whether effectiveness in reducing the risk of AO differed between CHX rinse and CHX gel, all of the CHX rinse trials were analyzed in one group, whereas all of the CHX gel trials were analyzed in a separate group. The results are summarized in Figure 4. No significant subgroup difference was observed (*I*^2^ = 53.7%, *p* = .14).

#### Chlorhexidine rinse versus chlorhexidine gel

Three trials compared the incidence of AO in the CHX rinse and CHX gel groups (Abu-Mostafa et al., 2015; Hita-Iglesias et al., 2008; Younus et al., 2014). The results of the meta-analysis showed a significantly lower incidence of AO in patients treated with CHX rinse than those treated with CHX gel (RR = 0.61, 95% CI [0.39, 0.95]; table not shown because of space limitations and may be obtained by contacting the corresponding author). No heterogeneity was observed across trials (*I*^2^ = 41%, *p* = .18).

## Discussion

In this study, using either CHX rinse or CHX gel after molar extraction was found to reduce the risk of AO. The subgroup analysis showed no difference between the CHX rinse trials and CHX gel trials in terms of AO risk. On the other hand, a meta-analysis of the three trials that compared CHX rinse and CHX gel suggested that CHX rinse is better than CHX gel in preventing AO.

It seems confusing at the first glance when looking at the insignificant difference between subgroups of all CHX rinse trials and all CHX gel trials and the significant effects resulting from the comparison of CHX rinse and CHX gel. This strongly suggests the presence of a methodology problem. The RR of a trial is affected by many factors, one of which is the treatment received by the control group. For example, saline control likely affords a certain level of AO prevention, whereas the option of no treatment probably has no AO prevention effect. Phenomena such as this affect the results to some extent and, unfortunately, are unable to be statistically adjusted for in the current study design. On the other hand, the number of trials used in the meta-analysis comparing CHX rinse and CHX gel was relatively small. Nevertheless, CHX gel appears to offer a few practical advantages over CHX rinse. First, only one application of CHX gel is required after molar extraction, whereas several days of CHX rinse are required. This implies that no further prescription is required for patients treated with CHX gel. However, an at-home CHX rinse must be prescribed for patients following the CHX rinse protocol along with additional health education to ensure compliance. In terms of cost, the market price of a 200-ml bottle of CHX rinse is approximately US$3. CHX rinse may be used by a single patient only because of hygienic concerns. By comparison, the market price of a 50-ml tube of CHX gel is approximately US$23.3. However, the gel can be used by 25 patients in a clinic (presuming 2 ml/patient) at a cost of less than US$1 per patient (price information obtained from the National Health Insurance, Taiwan). Certainly, the way that CHX products are applied affects their actual efficacies. For example, a recent RCT showed that irrigation of the molar surgical site with CHX had a better AO prevention effect than simply rinsing the mouth with CHX (Cho et al., 2018). Moreover, reducing the risk of AO reduces the need for return clinic visits because of extraction complications. In addition to benefits of treatment, side effects should be considered when evaluating treatment efficacy. Staining and bitter taste are two common side effects of CHX (Flötra et al., 1971; Tilliss, 1999) that have been at least partially addressed in newer CHX products (Raszewski et al., 2019). Practitioners may provide or recommend choices of CHX products to maximize their usage while minimizing their side effects and maintaining the targeted prophylactic effects. Another issue that should be noted is the potential that patients may exhibit an allergic reaction to CHX. A patch test involving 7,610 general dermatology patients using CHX digluconate (0.5% aqueous) revealed a 0.47% positive reaction in the sample (Liippo et al., 2011). Although the actual situation of CHX allergy in dentistry remains to be investigated (Pemberton & Gibson, 2012), precautions should be taken, especially among patients with a history of CHX contact allergy.

The trials included in our analysis showed considerable heterogeneity because of various backgrounds and clinical factors. Although most of the trials reported information on age and gender, which are known factors attributable to AO, some trials did not provide clear information on these basic variables. Whether all of the included trials examined young adults as revealed by most of the reported information could not be confirmed. Moreover, although we restricted the trials to molar extractions, we did not restrict the diagnostic criteria and molar extraction methods or narrow the target to a specific tooth (e.g., the third molar), nor did the standard used to diagnose AO. In addition, the experimental (i.e., methods of CHX rinse or CHX gel use) and control methods differed in the included trials. Other factors such as working experience and analgesics use that may affect the outcomes of surgery varied across the trials.

Our meta-analysis had some limitations. First, although molar extraction is a routine dental procedure worldwide, the number of RCTs related to AO and CHX rinse or gel is relatively low. Second, the included trials were conducted in a limited number of countries. Because overall oral health may vary across populations, the AO risk revealed in our results may differ slightly from the real-world situation in clinics.

### Implication for Practice

Both CHX rinse and gel treatments may be used after molar extraction to reduce the risk of AO. Although this measure improves health outcomes and dental health service quality, it only increases the treatment costs slightly. The findings of this review support the use of CHX rinse or gel in molar extraction surgery, whether covered by national insurance or self-pay. For convenience and affordability, CHX gel applied immediately after molar extraction by the dentist is recommended. The at-home CHX rinse is recommended as long as the dental clinic is able to provide appropriate health education and case management support.
